# Modified Screen-Printed Potentiometric Sensors based on Man-Tailored Biomimetics for Diquat Herbicide Determination

**DOI:** 10.3390/ijerph17041138

**Published:** 2020-02-11

**Authors:** Ayman H. Kamel, Abd El-Galil E. Amr, Nashwa S. Abdalla, Mohamed El-Naggar, Mohamed A. Al-Omar, Abdulrahman A. Almehizia

**Affiliations:** 1 Chemistry Department, Faculty of Science, Ain Shams University, Abbasia, Cairo 11566, Egypt; anoooosh311@gmail.com; 2 Pharmaceutical Chemistry Department, Drug Exploration & Development Chair (DEDC), College of Pharmacy, King Saud University, Riyadh 11451, Saudi Arabia; malomar1@ksu.edu.sa (M.A.A.-O.); mehizia@ksu.edu.sa (A.A.A.); 3 Applied Organic Chemistry Department, National Research Center, Dokki, Giza 12622, Egypt; 4 Chemistry Department, Faculty of Sciences, University of Sharjah, Sharjah 27272, UAE; m5elnaggar@yahoo.com

**Keywords:** diquat dibromide (DQ) solid-contact ISEs, poly(3,4-ethylenedioxythiophene) (PEDOT), screen-printed, molecularly imprinted polymers (MIPs), organic pollutant

## Abstract

Screen-printed platforms integrated with molecularly imprinted polymers (MIP) were fabricated and characterized as potentiometric sensors for diquat (DQ). The synthesized MIP beads were studied as sensory carriers in plasticized poly(vinyl chloride) membranes. The sensors were constructed by using poly(3,4-ethylenedioxythiophene) (PEDOT) as solid-contact material to diminish charge-transfer resistance and water layer potential. Conventional ion-selective electrodes (ISEs) with internal filling solution were used for comparison. The designed electrodes showed near Nernstian slopes of 28.2 ± 0.7 (r² = 0.999) over the concentration range of 1.0 × 10^−6^–1.0 × 10^−2^ M with the limit of detection 0.026 µg/mL over the pH range 4.2–9.0. The electrode exhibited good selectivity for diquat cations over a large number of organic and inorganic cations. The sensor was successfully introduced for direct measurement of diquat content in commercial pesticide preparations and different spiked potato samples. The results showed that the proposed electrode has a fast and stable response, good reproducibility, and applicability for direct assessment of diquat content. The proposed potentiometric method is simple and accurate in comparison with the reported HPLC methods. Besides, it is applicable to turbid and colored sample solutions.

## 1. Introduction

Pesticides and herbicides are extensively used in agriculture, forestry, and domestic activities for controlling pests. This rapid increase in their use can cause a real threat to the environment and human health. So, a highly restricted control must be followed to avoid unacceptable levels of these contaminants from entering the water environment, thus influencing the food chain of humans and animals [1].

Quats, a group of quaternary ammonium salts, is considered a particularly uneasy type of herbicide [2], attributed to their physico-chemical properties, which lags the known multi-residue methods from their quantification. Diquat (1,1′-dimethyl-4,4′-bipyridilium dibromide) is one of the most widely used herbicides. It is used for controlling aquatic weed and pre-harvesting desiccation of potato vines, carrots, onions, vines etc., and seed crops (including rice, peas, clover, rape, beans, maize, etc.) [3]. In addition, it holds the largest share of the global herbicide market until recently overtaken by glyphosate. This chemical type of herbicide—a bipyridyl—is shared with few other pesticides. Due to its high solubility (about 620 g/L at 25 °C), diquat (DQ) in fact a potential contaminant of waters [4]. It also sticks tightly via its doubly charged cation to the mineral anions present in the soil sediments for long periods without leaching to the groundwater [5]. The maximum contamination levels (MCL) for diquat in drinking water should not exceed 20 μg/L as stated by the United States Environmental Protection Agency (USEPA) [6]. The repeated exposures to DQ may result in skin irritation, sensitization, or ulcerations [7]. Chronic exposure may lead to cataract formation [8]. To be mentioned, diquat products contain the carcinogen ethylene dibromide (EDB) as a trace impurity. The occupational exposure limit for EDB should not exceed 0.13 ppm during any 15-minute sampling period as recommended by The National Institute for Occupational Safety and Health (NIOSH) [9]. So, in order to minimize risks associated with DQ use, there is an uprising need for a fast and reliable method for its quantification.

Different analytical methodologies have been reported in the literature for the determination of DQ different in real samples. These methods included voltammetric techniques [10,11,12,13,14,15,16], spectrophotometry [17,18], spectrofluorimetry [19,20,21], capillary electrophoresis (CE) [22,23], mass spectrometry coupled to either liquid chromatography [24,25,26,27] or HPLC [28], and gas chromatography [29]. Despite the fact of high sensitivity and selectivity of the above-mentioned analytical methods, the inconsistent results obtained suggested inconvenience with the chromatographic and/or mass spectrometric procedure for the quantification of DQ. To be noted, also is the sophisticated above-mentioned procedures, starting from extraction solvent composition, appropriate temperature, sample extract filtration for its pre-treatment for laboratory use, so as to obtain acceptable results. The ultimate goal is designing a simple, affordable, and easy to manipulate tool for DQ trace detection in real samples. In this context, potentiometric ISEs can be considered a good alternative because of their fast response, ease of automation, and applicability to turbid and colored matrices. As far, very few potentiometric sensors were reported for diquat monitoring [30,31,32,33].

Finding new designs for potentiometric ion-selective electrodes is the focus of attention of researchers working in this field. These new designs have the advantages of cost-effectiveness, ease of miniaturization and modification, robust and simple to be automated. Screen-printed platforms are examples of these new architectures. They are extensively used due their features such as no regeneration of the surface is required and highly reproducible geometric area for all electrodes. These interesting advantages can enhance the selectivity of the electrode and minimize any poisoning that can occur for the electrode surface [15].

Solid contact screen printed potentiometric sensors are stepping forward at a remarkable pace for trace level detection. They have attracted great attention over the past decades due to their simple planar design (no internal filling solution), low cost, and low equipment requirements. Carbon nanomaterials as ion-to-electron transducers were used for the fabrication of a high-performance and long-life solid state ISEs [34,35,36]. They are characterized by their high surface area, enhanced conductivity, and high ability to play the role of ion-to-electron transducer when mixed with ion sensing membrane or used as an electron conductor in solid state ISEs [37].

Molecularly-imprinted polymers (MIPs) are selective artificial receptors for a wide range of different templated molecules. Integration of MIPs in the fabrication of potentiometric ISEs exhibit a great attention to shift the view of using non-affordable ionophores, which are limited by their high cost, or using ion exchangers, which afford poor selectivity. Man-tailored polymers are characterized by many advantages such as high thermal stability, ease of preparation, and cost- effectiveness [38]. Recently, potentiometric ISEs based on MIPs as sensory elements have been fabricated for different templated organic molecules [39,40,41].

In this work, we present miniaturized planar potentiometric ISEs modified with poly(3,4-ethylenedioxythiophene) (PEDOT) as solid-contact material for selective detection of diquat (DQ) herbicide. The addition of PEDOT/PSS into the diquat-selective membrane enhanced the hydrophobicity and capacitance with considerable potential stability, which was tested by electrochemical impedance spectroscopy (EIS) and constant-current chronopotentiometry techniques. For comparison, liquid-contact ISEs were also prepared and characterized, then compared with the solid-contact ISEs. The proposed ISEs revealed a high sensitivity and selectivity for potentiometric monitoring of diquat in soil samples and determination in commercial herbicidal formulations.

## 2. Materials and Methods

### 2.1. Chemicals and Reagents

Diquat dibromide monohydrate, paraquat dichloride dehydrate, mepiquat chloride, chlormequat chloride, ethylene glycol dimethacrylate (EGDMA), methacrylic acid (MAA), benzoyl peroxide (BPO), poly (3,4-ethylenedioxythiophene)/poly(styrenesulfonate) (PEDOT/PSS), sodium tetrakis [3,5 bis (trifluoromethyl) phenyl] borate (NaTFPB), and acetonitrile were obtained from (Sigma, St. Louis, MO, USA) and used as received. Dioctyl phthalate (DOP), high molecular weight poly (vinyl chloride) (PVC), were obtained from Fluka AG (Buchs, Switzerland). Tetrahydrofuran (THF) was freshly distilled prior to use. All chemicals were of analytical grade and were used without further purification. Bi-distilled de-ionized water (BDW) was used throughout the work. Reglone 200 SL used for pesticide technical formulation was purchased from Syngenta Company (Cairo, Egypt) to make a 31.8 w/w% soluble liquid (SL) of diquat dibromide.

A (10^−2^ M) stock solution of DQ was prepared by dissolving 0.362 g of pure diquat dibromide monohydrate in 100 mL distilled water. Diluents (10^−2^–10^−8^ M) of DQ were preserved in brown bottles.

### 2.2. Apparatus

All potentiometric measurements were carried out at ±25 °C using an Orion pH/mV meter (model SA 720, Cambridge, MA, USA). Selectivity measurements were carried out using the so called “modified separate solution method (MSSM)” [42]. Fourier-transform infrared spectroscopy (FT-IR) measurements were carried out using FT-IR spectrometer (Alpha II, Bruker ABCO, Cairo, Egypt) using the attenuated total reflection (ATR) technique. Chronopotentiometry measurements of the screen-printed electrodes (SPE) were measured using Metrohom potentiostat/galvanostat (Autolab, model 204) purchased from Metrohom Instruments (Herisau, Switzerland). These tests were carried out in 10^−4^ M diquat solution using a conventional three electrode system including an ISE working electrode, Ag/AgCl (3 M) as the reference electrode, and a Pt wire as the counter electrode.

### 2.3. Man-Taillored MIPs Synthesis

In a 25 mL glass capped vial, diquat (DQ) (temblate), methacrylic acid (MAA) (monomer), and ethylene glycol dimethacrylate (EGDMA) (cross-linker) (in the ratio of 0.5:3:3 mmol) were dissolved in the porogenic solvent acetonitrile (15 mL). Free radical initiator benzoyl peroxide (BPO, 60 mg) was added, followed by passing a flow of N_2_ gas in to the mixture for 10 min to remove any dissolved oxygen. The solution is then sonicated for further 10 min to ensure solution homogeneity. The glass-capped vial was then immersed in a constant temperature oil bath for 18 h, preset at 75 °C. The control non-imprinted polymer (NIP) was prepared in a similar way as mentioned above, without involvement of the template molecule. The MIP was rendered void of the template by means of soxhlet extraction using methanol/acetic acid (8/2, v/v) and methanol, ascertained by the zero absorption of diaquat using a Shimadzu UV/VIS spectrophotometer (Model UV-1601), and by the Fourier-transform infrared spectroscopy (FT-IR). A 24 h period at ambient temperature was a sufficient time for the polymer to dry.

### 2.4. Screen-Printed Design and Sensor Fabrication

**Figure 1:**
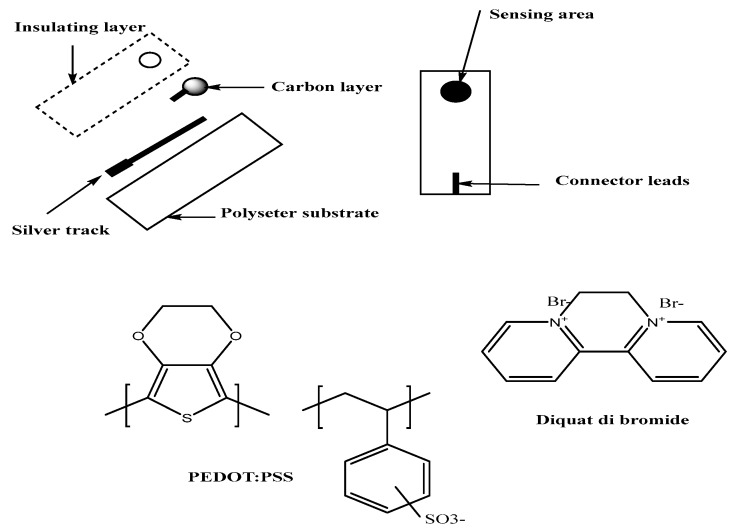
Scheme for screen printing of silver/carbon/PEDOT:PSS—diquat electrode.

The screen-printed platforms (5 × 30 mm) were designed and fabricated manually using a polyester sheet (~200 μm thick) as a substrate for electrode printing. Firstly, silver conductive ink was used to print the conducting track for the working electrode. Secondly, printing of carbon conductive ink at the end of the conducting track is done to form the sensing area of the working electrode. The polyester film, after the printing step, was heat cured at 150 °C for 15 min in a pre-heated oven. Finally, the electrodes were covered with an insulating tape leaving a rectangular area (3 × 3 mm) for defining the electrode sensing area as well as the connecter leads, forming a protective layer over the electrode tracking during analysis. Figure 1 shows the final fabricated screen-printed platforms which were further used for electrochemical analysis. To the carbon screen-printed platform (C/SPE), 10 μL of PEDOT/PSS was added to the conductive carbon using drop casting method and left to dry. This acts as the solid contact between the ion-sensing membrane (ISM) and the carbon conductor. The sensing membrane was prepared by dissolving 100 mg of the components in 2 mL THF as follow: (6.0 wt %) MIP or NIP, (1.0 wt %) (NaTFPB), (31.2 wt %) PVC, and (61.3 wt %) DOP. Then, 15 µL of the membrane cockatiel was added via drop-casting over the PEDOT/PSS layer pertain the sensing membranes. The sensors were left to dry for at least 6 h. Prior to usage, a 2 h soaking in 10^−3^ M DQ is convenient.

### 2.5. Diquat Determination in Real Samples

Analysis of real samples using the pre-designed screen-printed electrodes was checked towards their applicability as a diagnostic device for diquat monitoring in different commercial formulations and potato samples. Then, 10 g of a homogenized potato samples was placed into a 50 mL Teflon centrifuge tube. Fortified samples were prepared by spiking aqueous standards into the pre-weighed sample followed by 30 min equilibration. Then, 10 mL of 50:50 methanol/0.1 M HCl in water as an extraction solution was added to the sample. The mixture was shaken vigorously for 2 min by hand and heated in a water bath at 80 °C for 15 min. The supernatant was then analyzed using the proposed potentiometric method. Prior to HPLC analysis, the supernatant was filtered using a 45 micron PTFE syringe filter. A 500 μL portion of the filtered sample was diluted to 1.0 mL with acetonitrile prior to HPLC analysis.

In addition, DQ was analyzed in commercial agricultural formulations. Then, 0.5–1.0 mL of (Reglone 200 SL, Syngenta Company (Cairo, Egypt), 31.8 w/v% soluble liquid (SL) of diquat dibromide) formulations were diluted to 100-mL and their potential readings were used to look for their corresponding concentration along a calibration plot prepared from (10^−2^ to 10^−7^ M) standard diquat dibromide solutions.

## 3. Results and Discussions

### 3.1. Characterization of MIP Particles

**Figure 2:**
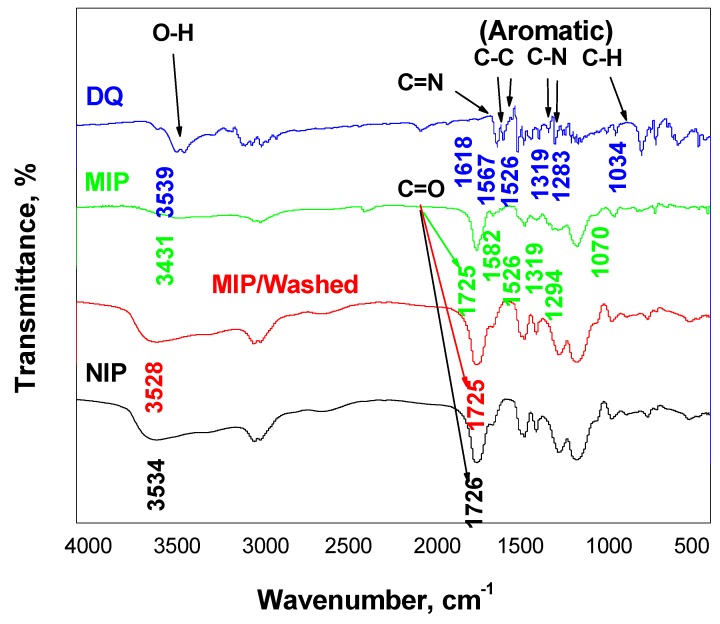
FT-IR spectra for: (**a**) diquat; (**b**) diquat /MIP, (**c**) MIP/washed and (**d**) NIP beads.

The imprinting process of diquat was examined using Fourier-transform infrared spectroscopy (FT-IR) technique and the measurements were carried out using the FT-IR spectrometer (Alpha II, Bruker ABCO, Cairo, Egypt) using the attenuated total reflection (ATR) technique. The FTIR spectra of diquat dibromide, non-washed MIP, washed/MIP and NIP nano-beads were shown in Figure 2. As mentioned in Figure 2a, a *υ*O–H stretch vibration peak appeared at 3390 and 3432 cm^−1^, arising from the water molecule in the hydrated diquat dibromide molecule. υC–H stretch vibration peaks appeared at 3049–3006 and 2949 cm^−1^ assigned for aromatic C–H. υC=C stretch vibration peaks appeared at 1572 and 1526 cm^−1^ assigned for aromatic C–C. υC=N stretch vibration peak appeared at 1618 cm^−1^. Further, almost all of these peaks already present in the FT-IR spectrum of the non-washed MIP were either in the same position or shifted as presented in Figure 2b. As shown in Figure 2c, the washed/MIP exhibited an O–H peak at 3528 cm^−1^. This is very close to the peak assigned to the O–H present in NIP beads (3543 cm^−1^) (Figure 2d). All FT-IR data were tabulated in Table 1. From the above-mentioned data, the imprinting of diquat was carried out successfully and the complete removal of it from the backbone of MIP was also achieved.

### 3.2. Potentiometric Characteristics of the Proposed Sensors

**Figure 3:**
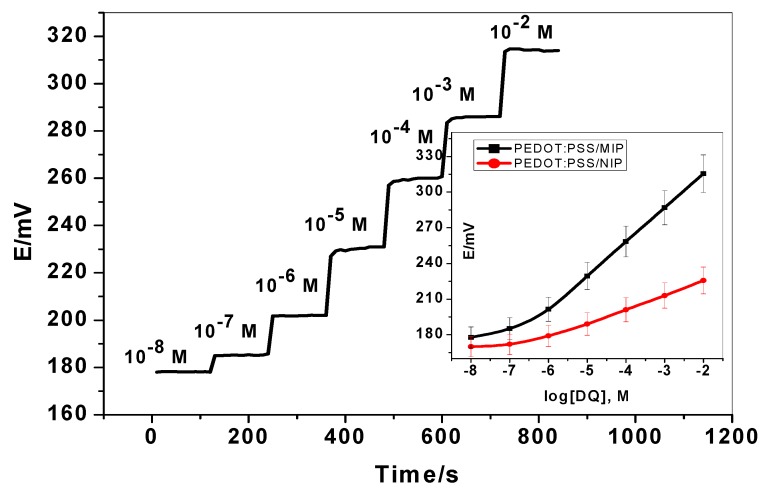
Potentiometric response curves obtained screen-printed ISEs integrated with MIP.

Sensitivity and linearity of PEDOT:PSS-based diquat-ISE were evaluated by measuring the potential in aqueous diquat solutions of different concentrations varying from 10^−8^ to 10^−2^ M. As shown in Figure 3, by increasing the concentration of diquat, the potential of PEDOT:PSS-based diquat-ISE was recorded as a function of time. The sensor revealed a Nernstian response across a linear range of 10^−6^–10^−2^ M with a slope of 28.2 ± 0.7 mV/decade (R^2^ = 0.999) and a detection limit of 0.026 µg/mL. For contrast as a control, diquat-ISEs based on NIP beads were also tested. These sensors possessed a linear range from 5.0 × 10^−6^–1.0 × 10^−2^ M of diquat solution, and the slope of calibration curve is computed to be 12.1 ± 0.5 mV/decade (R^2^ = 0.997) with a detection limit 0.05 µg/mL. All performance characteristics of the proposed sensors are presented in Table 2.

The dynamic time response of the proposed sensors was tested over the concentration range 10^−8^–10^−2^ M as shown in Figure 3. It was noticed that at concentrations below 10^−5^ M, the response time of PEDOT:PSS/MIP-ISE is lower than 10 s. At concentrations higher than 10^−5^ M, the sensors attain the equilibrium after 15 s. All these suggest that PEDOT:PSS/MIP-ISE possesses a better analytical performance, which is attributed to the function of PEDOT:PSS substrate for effectively transferring the ionic signal generating at the diquat-ISM into the electronic signal of the electrode.

Influence of the pH on the potential response of PEDOT:PSS/MIP-ISE and PEDOT:PSS/NIP-ISE was tested for (1 × 10^−4^ and 1 × 10^−3^ M) diquat solutions. It can be seen that the sensors revealed a stable potential over the pH range 4.2–9.0 and 4.5–9.0 for PEDOT:PSS/MIP-ISE and PEDOT:PSS/NIP-ISE, respectively. At pH <4, there is an observed potential drift due to H^+^ ion interference. On the other hand, the potential declined gradually at pH values >9.5. This can be attributed to the hydrolysis of diquat at this alkaline solution [43].

The selectivity coefficient (K^Pot^_I,J_) for PEDOT:PSS/MIP+NaTFPB was evaluated using the modified separate solution method (MSSM) [42]. The selectivity values are tabulated as the negative logarithm in Table 3. The obtained small selectivity coefficient values can be explained on the basis of the high selectivity of the proposed sensor towards diquat against the studied interfering pesticides and inorganic ions. Evidently, PEDOT:PSS/MIP+NaTFPB exhibited excellent selectivity towards diquat over paraquat, cyromazine, dinotefuran, acetamipride, Na^+^, K^+^, and Ca^2+^ ions. The obtained results reflected an enhanced selectivity for the proposed sensor and offered a great potential for trace-level monitoring of diquat in environmental samples.

The influence of light on the potential stability was measured by immersing the proposed PEDOT:PSS/MIP+NaTFPB ISE in 10^−5^ M diquat while turning on and off the ambient light. No obvious potential drifts were observed, demonstrating that the PEDOT:PSS-based solid contact has no light sensitivity. This result confirms that the PEDOT:PSS as the solid contact is superior to conducting polymers which is very sensitive to light.

### 3.3. Long-Term Potential Stability

**Figure 4:**
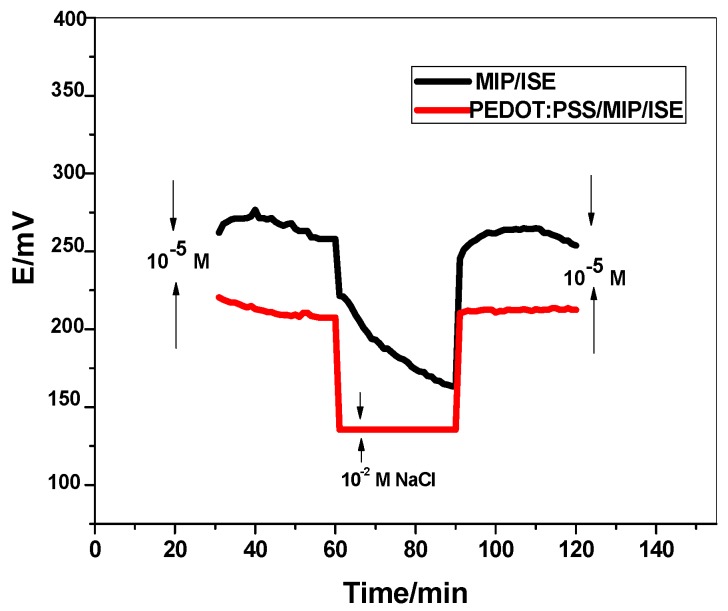
Water-layer tests for the diquat-ISE with and without PEDOT:PSS as solid contact transducer.

The presence of the water-layer between the transducer layer PEDOT:PSS and the ion-sensing membrane (ISM) could generate a severe potential drift for the ISE. So, the water-layer test is performed to distinguish the existence of this layer. This test was carried out by recording the potential of the proposed electrode when a solution of 10^−5^ M diquat was replaced by 10^−2^ M NaCl, then changed back to 10^−5^ M diquat again. As shown in Figure 4, PEDOT:PSS/MIP+NaTFPB ISE exhibits the characteristic EMF overshoot, which is usually seen when going back to the diquat solution. This suggests the absence of water-layer in the PEDOT:PSS/MIP+NaTFPB ISE. Moreover, long-term stability of PEDOT:PSS/MIP+NaTFPB and MIP+NaTFPB ISEs was evaluated via the potential response at the final part of the water-ayer test (Figure 4). The potential drift obtained was 0.11 and 3.3 mV/h for PEDOT:PSS/MIP+NaTFPB and MIP+NaTFPB ISEs, respectively. This indicates that addition of the PEDOT:PSS layer could enhance the potential stability of the proposed electrode and reflects its high lipophilicity character.

### 3.4. Short-Term Potential Stability

**Figure 5:**
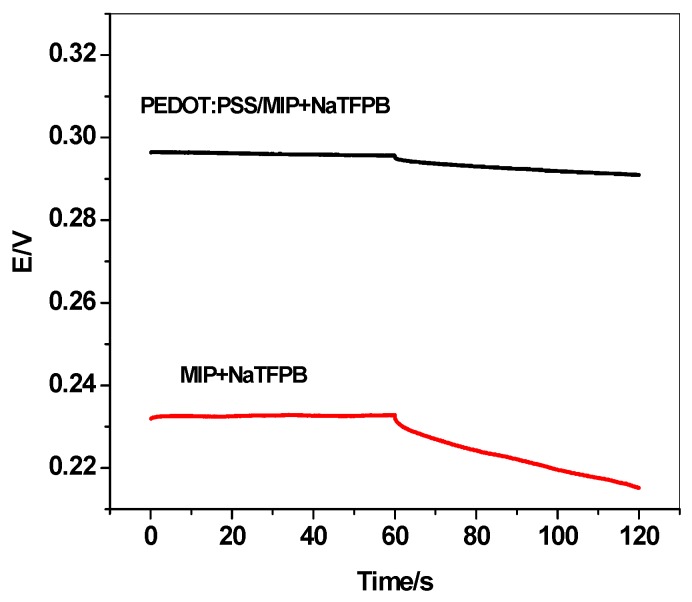
Chronopotentiograms (applied current: ±1 nA for 60 s) for solid-contact diquat ISEs.

Short-term potential stability was checked and estimated using the reverse chronopotentiometric technique. Typical chronopotentiograms for PEDOT:PSS/MIP+NaTFPB and MIP+NaTFPB ISEs were presented in Figure 5. After applying a ±1 nA current to the tree electrode cell, the overall resistance (*R_t_*) of the electrode was calculated using the potential shift in the response in accordance to Ohm’s law *R=E/I*. The total resistances were found to be 0.5 and 0.62 MΩ for PEDOT:PSS/MIP+NaTFPB and MIP+NaTFPB ISEs, respectively. Furthermore, the short-term potential stability was calculated through calculating the ratio of ΔE/Δt. The potential drift value was estimated to be 53.7 and 225.4 μV/s for PEDOT:PSS/MIP+NaTFPB and MIP+NaTFPB ISEs, respectively. The capacitance (C) of the fabricated electrodes was calculated via the fundamental capacitor equation, i.e., I = C × ΔE/Δt. The capacitance value for PEDOT:PSS/MIP+NaTFPB and MIP+NaTFPB ISEs was calculated as 18.8 ± 0.6 and 4.44 ± 0.3 μF, respectively. This reflects the significant importance of adding the PEDOT:PSS layer as a successful ion-to-electron transducer between the ISM and electronic conductor substrate.

### 3.5. Diquat Assessment

The proposed ISEs were introduced to assess diquat in commercial agriculture formulations containing diquat dibromide in addition to different potato samples. Reglone 200 SL, Syngenta Company (Cairo, Egypt), containing 31.8 w/v% soluble liquid (SL) of diquat dibromide) was taken as a commercial agriculture formulation for diquat assessment. Each sample was analyzed in triplicates. The results of analysis are shown in Table 4. These data were compared with results obtained by measuring diquat using the HPLC method as a comparative technique [44,45].

For the assessment of diquat in potato samples, the results obtained by both the proposed potentiometric method and the HPLC method as a comparative method were tabulated in Table 5. An *F*-test showed no significant difference at 95% confidence level between means and variances of the potentiometric and HPLC sets of results. The calculated *F*-values (*n* = 5) were found to be in the range of 1.6–4.8 compared with the tabulated value (6.39) at 95% confidence limit.

## 4. Conclusions

The present work entails the fabrication of a disposable, point of use screen-printed potentiometric ISEs for diquat detection. The trace level detection could be achieved by integrating a molecularly imprinted polymers (MIP) for diquat as a sensory recognition receptors and PEDOT:PSS as a solid contact transducer. This study reinforced the excellent characteristic of PEDOT:PSS as a good ion-to electron transducer for fabricating solid contact-ISEs. The sensors revealed fast response towards diquat with a potentiometric Nernstian slope and reasonable sub-Nernstian slope of 28.2 ± 0.7 mV/decade over the linear range of 1.0 × 10^−6^–1.0 × 10^−2^ M with a detection limit of 0.026 µg/mL. The sensor offered the advantages of reasonable selectivity over different common herbicides, good accuracy, and possible interfacing with automated systems. Advantages and limitations of many of the previously suggested potentiometric membrane diquat sensors are given in Table 6, for comparison. It can be seen that the sensors suggested in the present work have several inherent advantages over many of those previously described. The sensor was successfully applied in monitoring diquat in commercial agriculture formulations containing diquat in addition to different potato samples.

## Figures and Tables

**Table 1:** All characteristics FT-IR peaks for diquat, non-washed MIP, washed MIP and NIP beads.

Peaks	Diquat Dibromide Hydrate	Non-Washed MIP	Washed-MIP	NIP
υOH	3390, 3432	3431	3528	3543
υC–H stretch (aromatic and aliphatic)	3049–3006, 2949	2988–2955	2988–2956	2990–2955
υ-C=X stretching (X=N or O)	1618 C=N	1725 C=O	1726 C=O	1725 C=O
υC-C	1572, 1526	1582, 1526	-	-
C–N stretch aromatic amines	1319	1319	-	-
C–N stretch aromatic amines	1283	1294	-	-
C-H in-plane Bending (Aromatic)	1034	1070	-	-
=C-H in-plane bending	939	944	959	960
C-H bending (out-of-plane)	793	791	755	756
C-H bend Alkene	711	714	718	705
C-H bending (out-of-plane) Aromatic ring	643	645	-	-

**Table 2:** Performance potentiometric characteristics of diquat ISEs.

Parameter	PEDOT:PSS/MIP+NaTFPB	PEDOT:PSS/NIP+NaTFPB
Slope (mV/decade)	28.2 ± 0.7	12.1 ± 0.5
Correlation coefficient (r^2^)	0.999	0.997
Detection limit (µg/mL)	0.026	0.05
Linear range (M)	1.0 × 10^−6^–1.0 × 10^−2^	5.0 × 10^−6^–1.0 × 10^−2^
Working pH range (pH)	4.2–9.0	4.5–9.0
Response time (s)	<15 s	~15
Repeatability (% mV)	0.9	1.2
Reproducibility (% mV)	1.1	0.9
Accuracy (%)	99.2	98.7

**Table 3:** Potentiometric selectivity coefficients (log K^pot^_I,J_) of PEDOT:PSS/MIP+NaTFPB ISE.

Sensor	* Log K^Pot^_I,J_						
	Paraquat	Cyromazine	Dinotefuran	Acetamipride	K^+^	Na^+^	Ca^2+^
PEDOT:PSS/MIP+NaTFPB	−3.5 ± 0.2	−3.6 ± 0.4	−5.1 ± 0.7	−5.01 ± 0.2	−6.1 ± 0.4	−6.2 ± 0.7	−6.7 ± 0.2

* Mean value obtained from three corresponding pairs of concentrations of diquat and the respective interfering ions in the Nernstian response range ±standard deviation.

**Table 4:** Potentiomeric assessment of diquat in commercial agriculture formulation.

Commercial Product	Label (w/v%)	* Found			
		Potentiometry	RSD, %	HPLC [44,45]	RSD, %
Reglone 200 SL, Syngenta Company (Cairo, Egypt)	31.8	29.6 ± 1.1	93.1	30.3 ± 0.5	95.3

* Average of 3 measurements.

**Table 5:** Potentiometric assessment of diquat in some potato samples.

Sample	Amount Spiked, (µg/g)	* Amount, (µg/g)		*F*-test ^a^
		Potentiometry	HPLC [44,45]	
1	0.1	0.12 ± 0.03	0.11 ± 0.03	1.6
2	0.5	0.45 ± 0.01	0.52 ± 0.04	3.5
3	0.7	0.65 ± 0.04	0.68 ± 0.03	4.8
4	1.0	0.92 ± 0.07	1.01 ± 0.06	2.7

* Average of 5 measurements. ^a^ Critical tabulated *F*-value (*n* = 6) = 6.39 at 95% confidence interval.

**Table 6:** General characteristics of some potentiometric diquat membrane sensors.

Ionophore	Electrode Type	Slope, mV/decade	Linear Range, M	Detection Limit, M	Working pH Range	Ref.
Diquat-phosphotungstate	Carbon paste	30.8	3.8 × 10^−6^–1.0 × 10^−3^	9.0 × 10^−7^	4.5–9.5	[31]
Diquat bis (tetra-4-chloro phenyl borate)	Polymeric PVC	30.0	4 × 10^−9^–3 × 10^−6^	-	2.0–11	[32]
Different crown ethers	Polymeric PVC	33–41	-	1.7 × 10^−6^–6 × 10^−6^	-	[34]
Dibenzo 30-crown 10	Liquid	32.6	-	-	1.2–8.5	[46]
diquat ion pair with tetraphenyl borate	membrane	28.8	-	-	1.2–8.5	
